# Collaborative approaches in sustainable and resilient manufacturing

**DOI:** 10.1007/s10845-022-02060-6

**Published:** 2022-12-05

**Authors:** Luis M. Camarinha-Matos, Andre Dionisio Rocha, Paula Graça

**Affiliations:** 1 School of Science and Technology and Uninova-CTS, NOVA University of Lisbon, Campus de Caparica, Caparica, 2829-516 Portugal; 2 Instituto Superior de Engenharia de Lisboa, Instituto Politécnico de Lisboa, Rua Conselheiro Emídio Navarro 1, Lisbon, 1959-007 Portugal

**Keywords:** Collaborative networks, Sustainable manufacturing, Resilience, Industry 4.0, Digital transformation, Industry 5.0, Sustainability indicators

## Abstract

In recent years, the manufacturing sector is going through a major transformation, as reflected in the concept of Industry 4.0 and digital transformation. The urge for such transformation is intensified when we consider the growing societal demands for sustainability. The notion of sustainable manufacturing has emerged as a result of this trend. Additionally, industries and the whole society face the challenges of an increasing number of disruptive events, either natural or human-caused, that can severely affect the normal operation of systems. Furthermore, the growing interconnectivity between organizations, people, and physical systems, supported by recent developments in information and communication technologies, highlights the important role that collaborative networks can play in the digital transformation processes. As such, this article analyses potential synergies between the areas of sustainable and resilient manufacturing and collaborative networks. The work also discusses how the responsibility for the various facets of sustainability can be distributed among the multiple entities involved in manufacturing. The study is based on a literature survey, complemented with the experience gained from various research projects and related initiatives in the area, and is organized according to various dimensions of Industry 4.0. A brief review of proposed approaches and indicators for measuring sustainability from the networked manufacturing perspective is also included. Finally, a set of key research challenges are identified to complement strategic research agendas in manufacturing.

## Introduction

During the last decade there has been a considerable evolution in the manufacturing sector as reflected in the Industry 4.0 / Industry 5.0 and digital transformation “movement” (Xu et al., 2021; Zhang et al., 2021a). The convergence of multiple new technologies that reached a point of maturity, and the political support for this “industrial revolution” led to the emergence of new organisational and managerial forms, the development of new processes, the notion of extended product-service systems, and the development of new business models (Camarinha-Matos et al., 2019).

At the same time, the manufacturing sector also faces the challenge of responding a growing societal demand for sustainability and social responsibility. Such demand is well reflected in the UN Agenda 2030 (United Nations, 2015), which establishes 17 goals for sustainable development. In this agenda, manufacturing has a key role, as expressed in the various sub-items of Goal 9, “*Build resilient infrastructure, promote inclusive and sustainable industrialisation and foster innovation*”. But it goes beyond that as other (indirect) references to manufacturing are found in various other goals of the Agenda, e.g., “*achieve higher levels of economic productivity through diversification, technological upgrading and innovation*”, “*double the global rate of improvement in energy efficiency*”, “*promote development-oriented policies that support productive activities, decent job creation, entrepreneurship, creativity and innovation, and encourage the formalisation and growth of micro-, small- and medium-sized enterprises*”, etc. Reflecting this trend, the term “sustainable manufacturing” (OCDE, 2021) is becoming quite relevant and receiving growing attention.

More recent discussions around the notion of Industry 5.0 (Breque et al., 2021; i-Scoope, 2022) and Society 5.0 (H-UTokyo Lab, 2020; Broeckaert, 2022) emphasize the need to focus on the aspects of sustainability, resilience, and human-centric systems.

In this context, and as initially identified in (Camarinha-Matos et al., 2010), there is a great potential in exploiting mutual beneficial synergies between the areas of sustainability science and collaborative networks. This earlier work was one of the first arguing that effective implementation of sustainability requires a wide collaboration among multiple stakeholders, pointing thus to a notion of co-responsibility. It was also emphasized that it is not possible to achieve sustainability by the effort of individual entities alone. Furthermore, collaborative networks have also identified as a “core enabler for Industry 4.0 and digital transformation” (Camarinha-Matos et al., 2017, 2019).

Consistent with these trends and the idea of a collaborative approach to sustainable manufacturing, this work is guided by the following research question:

### What is the role of collaborative networks in sustainable and resilient manufacturing?

This article, which is an extended version of a preliminary presentation at PRO-VE 2021 (Camarinha-Matos et al., 2021), focuses on identifying and categorizing relevant trends and examples to help understand the synergies among the areas of manufacturing, collaborative networks, and sustainability. The remainder of the article is structured as follows: in the next section, a set of base concepts are briefly summarized in order to give a basis for the following sections; the following section discusses the role and opportunities for the adoption of collaborative networks models, principles, and mechanisms in the various dimensions of Industry 4.0 and the complementary vision of Industry 5.0; the article continues with two sections describing the main trends found in literature and example projects, as well as approaches and indicators to measure sustainability. The main body of the article ends then with a proposal of contributions for a research agenda in this area; and finally, some conclusions are presented.

## Base concepts

In order to set a context for the discussion, some related basic concepts are briefly revisited in this section.

The notion of *sustainability* is typically analyzed under three perspectives: environmental, social, and economic (Camarinha-Matos et al., 2010). This notion involves considerable complexity, both due to its multi-dimensional nature, and also because it requires a difficult balance among objectives that are often conflicting and involves multiple stakeholders. When focusing specifically on manufacturing, various related terms are often used in the literature, including “sustainable manufacturing”, “industrial symbiosis”, and “circular economy”.

**Sustainable manufacturing** is a concept representing the “*integration of processes and systems capable to produce high quality products and services using less and more sustainable resources (energy and materials), being safer for employees, customers and communities surrounding, and being able to mitigate environmental and social impacts throughout its whole life cycle*” (Machado et al., 2020). A similar definition is offered in (OCDE, 2021), which further emphasizes the need for those processes to be “economically sound”. The same document illustrates well the three dimensions of sustainable manufacturing:

1. Environmental dimension, e.g., using environmentally sound materials and energy, minimizing waste and emissions, minimizing the use of hazardous substances, using energy and resources efficiently, protecting biodiversity;
2. Social dimension, e.g., ensuring good community relations, guaranteeing good working conditions, ensuring product safety, treating suppliers fairly, complying with the law, respecting human rights;
3. Economic dimension, e.g., contributing to the local economy, creating jobs, investing in infrastructures, driving innovation, paying taxes responsibly, generating sales and profits, combating bribery and corruption.

Despite all these conditions, the report also claims that moving to this new way of doing business creates value and can give companies a competitive advantage.

**Industrial symbiosis** corresponds to one specific implementation of sustainable manufacturing representing a “*process by which the wastes or by-products of an industry or industrial process become the raw materials for another*” (EGC, 2018). This notion naturally implies a collective effort by which a group of separate industries form a kind of collaborative business ecosystem to exchange materials, water, energy, and by-products (Baldassarre et al., 2019). This concept implies moving from a linear model of “take-make-dispose” to a circular model in which the waste of some processes is valorised as a resource for others.

**Circular economy** is a more general concept, which focuses on “*higher resource utilisation by recollecting and reusing components of products after their use is over*” (Pomponi & Moncaster, 2017). As such, it “*enables the reintegration of materials into production processes through their reuse, recycling, and recovery*” (Azevedo et al., 2010). From a traditional point of view, while the circular economy focuses on the entire economy, sustainable production may seem focused only on the manufacturing stage (Enyoghasi & Badurdeen, 2021). However, when we take the view of Industry 4.0, and more specifically the notions of extended and smart product, considering the entire product life cycle, the notions of sustainable manufacturing and circular economy overlap more.

The effective implementation of all these notions requires some form of collaboration between the actors involved, so the role of collaborative networks in supporting sustainable production deserves attention. Indeed, the usual notion of **collaborative network** as “*composed of a variety of entities – organizations people* and even smart machines *– which are largely autonomous, geographically distributed, and heterogeneous in terms of their operating environment, culture, social capital and goals… that collaborate to (better) achieve common or compatible goals*” (Camarinha-Matos et al., 2009) encompasses a comprehensive view of the interactions and inter-dependencies that exist between the multiple entities involved in a manufacturing system. Furthermore, the notion of business community or business ecosystem, as represented by the Virtual organization Breeding Environments, helps to achieve a better perception of *co-responsibility* of all involved actors regarding the challenges of sustainability (Camarinha-Matos et al., 2010). In this direction the notion of circular ecosystem has also been introduced (Konietzko et al., 2020).

On the other hand, contemporary manufacturing systems are increasingly exposed to a variety of disruptive events that may severely affect their operation. Such events may result from a large variety of factors, including natural disasters, pandemic situations such as COVID-19, terrorism, wars and political instability, climate change, economic crisis, demographic shifts, etc. (Chroust & Aumayr, 2017; Ramezani & Camarinha-Matos, 2019; Ivanov & Dolgui 2021). Such disruptive events appear to be increasing in frequency and in harmful effects, which may seriously affect the socio-economic dimensions of sustainability. As such, new terms became widely used:

**Resilience**, which is a characteristic of systems that, after a brief temporary change as a result of a disruption, can recover from such event and return to some acceptable state (not necessarily the same as before). Furthermore, the term **transformative resilience** (Dahlberg, 2015) is sometimes used to describe systems that resist to disruptions/shocks and not just conserve their structures, but rather “re-organize, reconfigure, restructure, and even reinvent themselves” (Ramezani & Camarinha-Matos, 2020) in response to disruptions.

**Antifragility**, which is a characteristic of systems that can absorb shocks / attacks and get better afterwards (Taleb, 2012). Thus, a property of systems that adapt to disruptive and volatile contexts, learn from experiences and incidents, and become stronger.

Alo related are recent discussions on viability, a notion that has been extended to encompass resilience and sustainability, as reflected in:

**Viable business ecosystems and supply networks**, which refer to systems that are dynamically adaptive and structurally changeable, to be agile, resilient, and able to survive at times of long-term global disruptions, in line with sustainability developments (Ivanov, 2020). This notion is also related to the earlier concept of Minimum Viable Ecosystem (Adner, 2013), which was less concerned with sustainability in general.

## Research method

In this work, we pursue the goal of understanding the synergies between the areas of sustainability, manufacturing and collaborative networks. For this purpose, we have adopted a mixed method, combining a systematic literature-based mapping study with case studies/experiences gained from various research projects.

The Systematic Literature Review (SLR) method has been applied in different research fields and aims at aggregating evidence through a “systematic, replicable, and transparent process” to synthetize research results and existing practices (Kitchenham et al., 2009). However, considering that we are not focused on a single area but rather interested in identifying synergies among three different areas, we had to follow a light version of SLR more focused on a mapping study.

In order to guide the SLR part the main research question was further sub-divided into related sub-questions: (1) How are collaborative networks aspects supporting sustainability in the various dimensions of manufacturing systems?, (2) Which performance assessment frameworks, metrics and indicators have been suggested for sustainable and collaborative manufacturing systems?

Well known indexing databases were used for search, namely Google Scholar, SCOPUS and Web of Science. Although aiming at including a good sample of reported results, we mainly focus on classification and mapping, rather than on performing a statistical analysis of existing empirical evidence (Petersen et al., 2015). The period 2017–2021 was the main target in order to capture recent results, but given the exploratory nature of the mapping exercise, in some cases it was relevant to trace back some lines of development and include some earlier references. Through a preliminary screening phase, only those works with a contribution to the mentioned research questions were retained for analysis.

Regarding direct experience collected from research projects, the authors have been involved in dozens of European and national projects with links to the addressed topics, but for illustration purposes only two of them are highlighted, illustrating the discussed issues in different dimensions of manufacturing systems. It shall be mentioned that many other projects are in fact considered through the analysis of publications that originated in such projects.

Complementarily, through discussion sessions and panels in a series of recent conferences (e.g. PRO-VE, INDIN, IoT, and DoCEIS), the authors collected feedback on the trends in sustainable and resilient manufacturing, which contributed to consolidating the findings presented in this article. For instance, a position paper (Camarinha-Matos at al., 2017) was presented, followed by discussions in focus groups and panels along the last four to five years. Most of the consensus achieved in those discussions has not been reported, with the exception of a summary on trends in IoT research, which nevertheless was not restricted to manufacturing (Camarinha-Matos & Katkoori, 2022).

In order to facilitate the aimed mapping and identification of synergies, next section introduces a classification of six dimensions of analysis for advanced manufacturing systems. This classification was first introduced in (Camarinha-Matos et al., 2021), but is discussed here under the perspective of analyzing the role of collaborative networks in sustainable manufacturing. These dimensions are later used to frame the findings on trends. It should be noted that among the few systematic reviews on sustainable manufacturing, such as (Jamwal et al., 2021), only the interrelationships between sustainability and manufacturing are considered, and the collaboration perspective that is central to our study is mostly missing.

We also hope that the identification of synergies among the considered areas will contribute to a better understanding and characterization of the next generation of collaborative networks. Typically, a business ecosystem or any other form of collaborative network does not just involve collaboration, but rather comprises a complex and dynamic combination of collaboration and competition. In these business communities, one can typically observe some form of “survival instinct” and shared vision that can lead members to align their activities and commitments and play mutually supportive roles, contributing to sustainability and viability. By further discussing these ideas, we hope to gain new insights into better organizational structures and governance principles that will likely contribute to more sustainable and resilient manufacturing ecosystems. Thus, this work is also guided by the general principles of strategic research roadmapping (Camarinha-Matos & Afsarmanesh, 2004), namely in terms of (i) analysis of baseline, (ii) identification of strategic visions, (iii) research gap analysis, and (iv) proposition of actions to complement existing research agendas, namely the ones mentioned later in the section on Research Challenges.

## Collaborative networks in advanced manufacturing systems

Several recent works have advocated the adoption of collaborative networks as one of the core enablers for Industry 4.0 and the associated digital transformation processes (Camarinha-Matos et al., 2017, 2019; Santos et al., 2021; Torn & Vaneker, 2019). Indeed, when we consider the various dimensions of the fourth industrial revolution, including both the manufacturing system and the product/service perspectives (Table 1), it becomes clear that we need to deal, in all these dimensions, with networks involving multiple actors, being organizations, people, smart machines, and intelligent systems, with different degrees of heterogeneity and autonomy. More than integration, we need to deal with smart, heterogeneous, and autonomous elements whose potential can be harnessed when we move from a focus on interoperability and control, to a context of negotiation, contracting, and sharing, which are characteristics of a collaborative environment.

**Table 1:** Typical dimensions of Industry 4.0 and digital transformation

Dimension		Brief description
**Manufacturing system perspective**	**1. Vertical integration** or networking of smart production systems	Integration of systems and processes at the various vertical layers of the enterprise, from shopfloor to the upper layers of engineering and business management. This integration facilitates real time data access and transparency, better supporting decision-making and agility.
	**2. Horizontal integration** through global value chain networks	Involves collaborative networking with suppliers, distributors, and other business partners. This integration facilitates smooth flow of information and materials along the supply chain, and thus collaboration among all involved stakeholders.
	**3. Acceleration of manufacturing**	Aims at optimization of manufacturing systems through the integration of the so-called “exponential technologies”, thus accelerating and making processes more flexible. This also involves collaboration with newcomer actors representing those technologies and the traditional actors of the manufacturing environment.
**Product/Service perspective**	**4. End-to-end engineering** or through-engineering across the entire value chain	Integration of all product-related engineering activities through the whole product life-cycle, namely from design/manufacture to disposal/recycling. It involves internal collaboration among multiple departments as well as external collaboration with stakeholders of the value chain and the customers.
	**5. Smart products & Digitalization of products and services**	Covering various sub-dimensions: (i) digital models of products (and even their digital twins), (ii) adding services to products, (iii) moving towards smart products (which include sensing, computing, and communication capabilities).
	**6. New business models and customer engagement**	Involving the emergence and development of novel business models taking advantage of digitalization and networking. Early examples include product-service systems, *glocal* enterprise, hybrid value chains, customer intimacy, etc., but other models are likely to emerge.

**Fig. 1:**
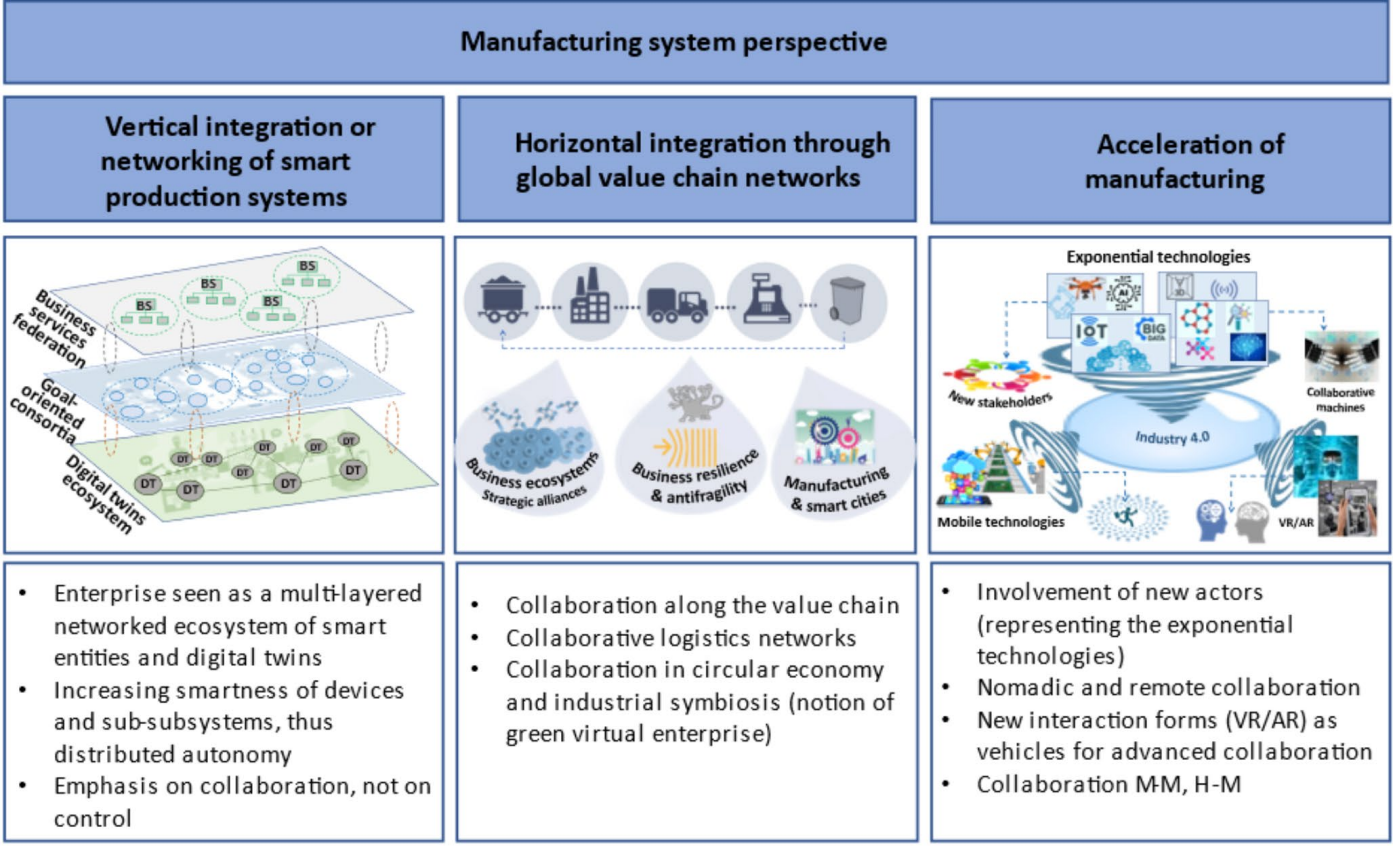
Examples of collaborative aspects in the manufacturing system dimensions

**Fig. 2:**
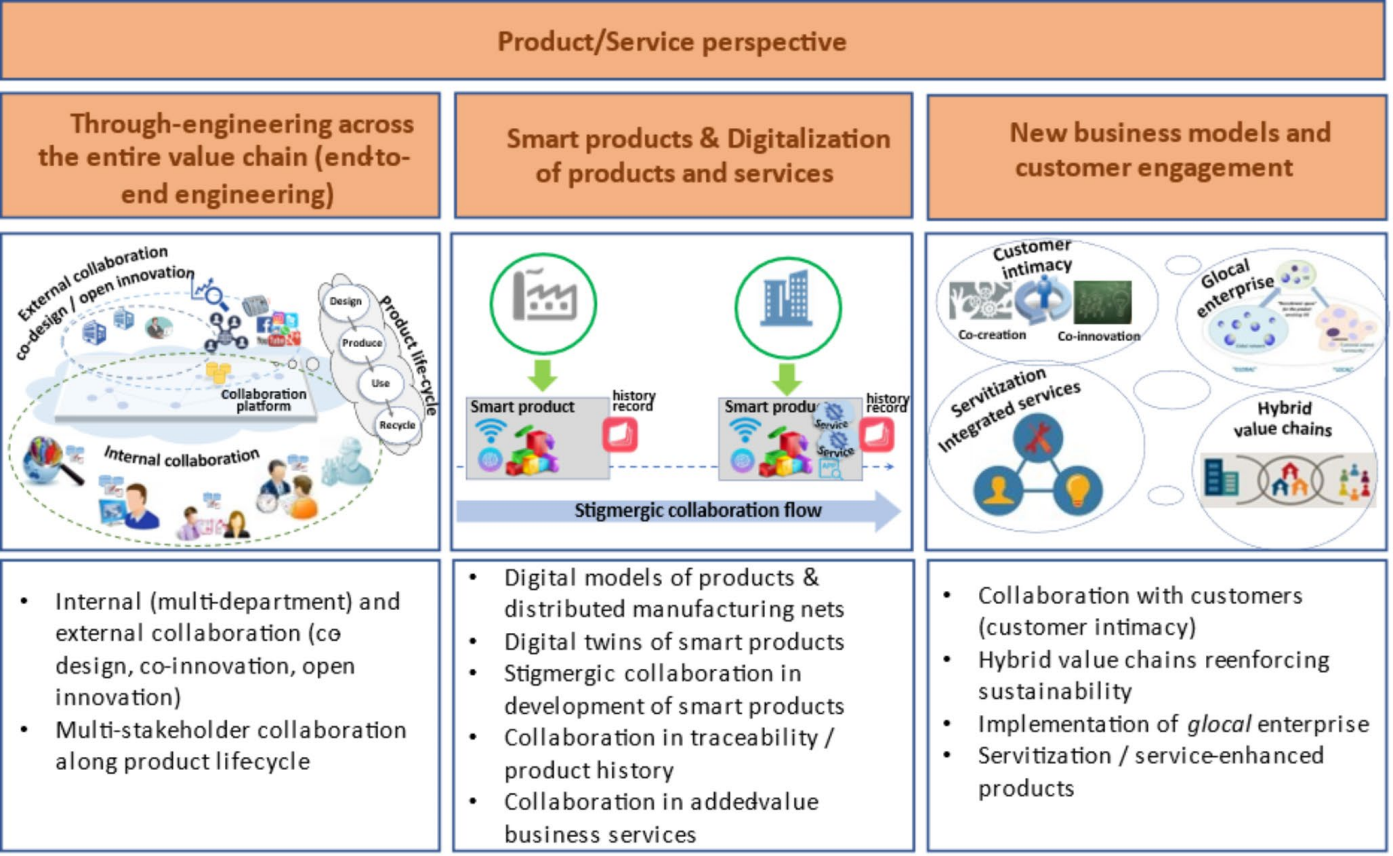
Examples of collaborative aspects in the product/service dimensions

Some highlights of this trend toward collaborative systems are illustrated in Figs. 1 and 2, which go far beyond the traditional view where networks were only considered in relation to value chains, and rather fundamentally influence all dimensions of Industry 4.0. The included examples do not intend to be a comprehensive list, but rather an illustration of the idea. In fact, it is too early to attempt a comprehensive categorization of aspects as many new ideas for the adoption of a collaborative perspective in this sector are still emerging.

Of particular relevance in vertical integration is the interconnection of the physical and the cyber worlds as reflected in the areas of Cyber-Physical Systems (CPS) and Internet of Things (IoT) (Oztemel & Gursev, 2020). As part of this integration, the representation of physical entities (e.g., shop floor equipment) in the cyber world led to the concept of “digital twin” (Lim et al., 2020). A digital twin constitutes a cyber “reflection” of a physical entity, being “synchronized” with that entity. As devices and machines become smarter, acquiring increased levels of autonomy, we can refer to smarter or cognitive digital twins, and the shopfloor can be seen as a collaborative ecosystem of digital twins. Thus, instead of monolithic manufacturing execution systems, we can think of dynamic consortia formation (of digital twins) according to the production needs. Similarly, at the upper levels of the enterprise, instead of large monolithic systems like the traditional ERP systems, we move to federations of (collaborative) services. This leads to a view of the enterprise as a multi-layered collaborative network ecosystem of smart components and people.

At the horizontal integration level, the aim is to bring all stakeholders in the value chain into closer relationships with each other. This involves networking of suppliers, manufacturers, distributors, service providers, product recycling entities, and even the customers. Collaboration not only contributes to an improved operation of the supply chain, but also helps to increase resilience and implement circular economy. As we move from large and centralized manufacturing facilities to smaller, distributed manufacturing units, there is a clear need to increase the networking and collaboration among such units. Visionary ideas such as personal or social manufacturing, inspired by the possibilities of additive manufacturing (e.g., through Fab Labs), and even the movement of “bring factories back to the cities” (Juraschek et al., 2016), require and fully embed the notion of collaborative networks in manufacturing.

In terms of the acceleration of the manufacturing dimension, we observe the “arrival” in the manufacturing sector of a number of new “actors” representing the so-called “exponential technologies” (Deloitte, 2015), which bring new ways of work, which require effective collaboration with the traditional manufacturing stakeholders. These new technologies also bring new forms of human-machine/system interaction (e.g. virtual reality, augmented reality, remote and mobile interaction), which give rise to new forms of collaboration such as nomadic collaboration, collaborative robotics, etc.

This view of a manufacturing system as being composed of multiple inter-related networks of autonomous or partially autonomous entities implicitly entails the notion of a *distribution of responsibilities* among these entities. Consequently, the issue of sustainability also needs to be viewed from a collaborative networks perspective, since multiple entities/sub-systems are *co-responsible* for the level of sustainability that can be achieved by the manufacturing system.

Regarding the product/service perspective, an overview is presented in Fig. 2. Similar to Fig. 1, the included examples are given only for illustration purposes.

With regard to the end-to-end engineering dimension, where the aim is to integrate all engineering activities involved in the entire product life-cycle, from design and manufacturing to disposal and recycling, both internal and external collaboration needs to be promoted. Internal to the manufacturing company, this involves collaboration among various departments (e.g., market research, product design, service design, production planning, and manufacturing departments). External collaboration involves suppliers and customers in co-design/co-innovation, and even involvement in social networks to better perceive market trends. The aspects of servicing during product use, and recycling at the end of the product life, expand the need for external collaboration.

Smart products and digitalization comprise two main sub-dimensions: elaboration of digital models of products (and services) and development of smart products. In other words, digitalization involves not only the creation of digital models of products, but also a progressive evolution towards smart products that embed computing, sensing, actuation, and communication capabilities. In particular, adding “memory tags” to products allows for recording of products’ history and thus facilitates tracking and traceability. Through these “history records”, we can envision a kind of “stigmergic collaboration” (Camarinha-Matos & Afsarmanesh, 2018) among the various stakeholders in the manufacturing value chain. This dimension is also related to the addition of business services to the physical product, as reflected in the terms “service-enhanced product”, “extended product”, or “product-service system”, whose effective implementation requires collaboration among multiple enterprises.

The intense networking and ongoing digital transformation processes induce the emergence of new business models. This includes, for instance, exploitation of closer customer relationships (“customer intimacy”), exploitation of global markets with adaptation to local/regional specificities/“flavors” (the notion of “*glocal* enterprise”), combination of non-profit and for-profit stakeholders to solve societal problems (notion of “hybrid value chains”), creation of new bigdata-related services, etc. In all these models, effective collaboration among multiple stakeholders is a key issue. Particularly during the COVID-19 pandemic, new ways of taking advantage of networked collaboration were noticeable in many sectors, reflecting some forms of transformative resilience or even antifragility.

In past literature, the role of collaborative networks in supporting sustainability has been widely addressed in relation to the “horizontal integration dimension”, namely in terms of circular economy and industrial symbiosis, or in relation to “new business models”, as we can find in some cases of hybrid value chains (Baldassarre et al., 2019; Azevedo et al., 2017). However, the issue has been less studied for the other dimensions, which justifies an effort to analyze existing trends and relevant examples to identify research gaps.

## Trends and examples

While Industry 4.0 has typically been more concerned with technology development and integration, some recent developments have begun to place some emphasis on solutions aimed at introducing more sustainable manufacturing practices, not only from a cost and profit perspective, but also considering the other two pillars of sustainability, namely social and environmental aspects. Although this trend towards more sustainable ecosystems is more frequently mentioned in recent literature, there is generally no assessment of how aspects of collaboration are directly or indirectly related to the improvement of these complex ecosystems. These distributed and complex systems imply more intense communication between actors to optimize the systems, whether from an economic, social and/or environmental perspective. Similarly, although the issues of resilience and antifragility have been around for some time (Ramezani & Camarinha-Matos, 2019), their positioning in the context of collaborative and sustainable manufacturing is still limited.

The association of a collaborative networks perspective with these systems is often not very explicit in the manufacturing literature. The exception is the case of the horizontal dimension, where networks of enterprises have been extensively studied. However, in many publications, it can be identified that collaboration is an essential aspect of the design and operation of the addressed complex environments. For example, we can find the implementation of shopfloors where machines collaborate with each other and/or with human operators. Another case widely observed in the literature is the optimization of distributed manufacturing systems where different plants, suppliers and transportation systems work together to optimize their operation as a whole. New products are also emerging with new features such as communication, sensing, computing, and cloud connectivity that allow manufacturers to extract data from products and remotely modify their functionality. These facets also bring a new impetus to the old idea of concurrent engineering, contributing to the fact that product design is done in collaboration between different departments in the company, including the customer, and using the product itself as a “means of interaction” in this process (some form of stigmergy). This new reality is becoming clear with the introduction of new emerging technologies such as Cyber-Physical Systems, Artificial Intelligence or Additive Manufacturing, which will force companies to apply new business models.

Table 2 shows a summary of representative studies focused on the development of sustainable manufacturing systems in which collaborative aspects are present. This table covers the infrastructure or manufacturing system perspective and presents examples of proposals that aim at increasing the sustainability of manufacturing systems through collaboration.

**Table 2:** **–** Examples of collaboration and sustainability aspects in the manufacturing system dimensions

	Economic	Social	Environmental
**Vertical Integration**	• ***Cost reduction & increase of productivity***: - Machine collaboration (Adamson et al., 2017; Zhou et al., 2018; Wang et al., 2020). - Collaborative robotics (Calvo & Gil, 2022). - Human-robot collaboration (Gualtieri et al., 2020; Lv et al., 2021). • ***Increase efficiency***: - Self-organization and shared resources (Li & Jiang, 2021). • ***Improve interoperability in collaborative automation***: - Semantic aware communication (Lu et al., 2020).	• ***Improve working conditions & reduce health problems***: - Human-robot collaboration (Renteria & Mozos, 2019; Gualtieri et al., 2020; Ansari et al. 2020; Lv et al., 2021; Poschmann et al., 2021). • ***Support short-term workforce displacement***: - Collaborative robotics & social impact (Calvo & Gil, 2022).	• ***Reduce energy & resources consumption***: - Shared factories (Li & Jiang, 2021). - Collaborative agent-based Cyber-Physical System and optimization engine (Raileanu et al., 2017). • ***Reduce waste & improve recyclability***: - Human-robot collaboration in circular economy (Renteria & Mozos, 2019; Poschmann et al., 2021).
**Horizontal Integration**	• ***Cost reduction***: - Auction-based and PSS-based logistics (Kang et al., 2021). - Blockchain in reducing transaction costs (Kang et al., 2021). - Collaborative strategies and eco-packages to minimize operational costs (Wang et al., 2021b). - Environmental collaboration and cost saving (Grekova et al., 2016). • ***Improve resource allocation***: - Collaborative resource allocation (Li et al., 2018; Upadhyay et al., 2021). • ***Increase resilience***: - Machine Learning & Data-driven simulation of supplier selection (Cavalcante et al., 2019).	• ***Increase social welfare and human rights***: - Collaboration, blockchain and social responsibility (Upadhyay et al., 2021). • ***Improve customer value***: - Green supply chain and supply risks (Lintukangas et al., 2016). • ***Increase resilience***: - Sustainable collaborative governance of supply chains (Wang & Ran, 2018).	• ***Reduce resources’ waste***: - Sharing spaces and machines (Wang et al., 2021). - Auction-based and PSS-based logistics (Kang et al., 2021). - Industrial symbiosis and waste management (Chen & Liu, 2021) • ***Reduce carbon footprint and energy consumption***: - Sustainable collaborative supply chains (Upadhyay et al., 2021; Glatt et al., 2021). - Methods to select suppliers for sustainable supply chains (Sarkis & Dhavale, 2015; Trapp & Sarkis, 2016; Wu & Barnes, 2016; Cavalcante et al., 2019).
**Acceleration of Manufacturing**	• ***Interoperability and integration of resources***: - Collaborative CPS in resource sharing (Adamson et al., 2017). - Semantic-aware CPS for machine-to-machine communications (Lu et al., 2020). • ***Collaboration in product design***: - Digital twins to support the design of products by different teams and at different stages (RTao et al., 2019). • ***Improve shared manufacturing***: - Digital Twin-driven and credit-based resources allocation (Wang et al., 2021). - Self-organizing agents (Li & Jiang, 2021). - Cloud-based manufacturing services ecosystem (Zhang et al., 2019). • ***Increase resilience***: - Machine learning in resilient supplier selection & delivery reliability (Cavalcante et al., 2019).	• ***Include customer in the process***: - Through additive manufacturing / 3D printing (Rayna et al., 2015; Turner et al., 2019). • ***Improve social aspects***: - Blockchain in collaborative distributed ecosystem & trust increase (Upadhyay et al., 2021). - Cyber-Physical-Social-connected and service-oriented manufacturing (Jiang et al., 2016). - Blockchain to handle cyber-credits among makers in “social manufacturing” (Leng et al., 2019). • ***Increase collaboration between humans and robots***: - Digital Twins in collaborative assembly (Lv et al., 2021).	• ***Reduce energy & resources consumption***: - Distributed manufacturing of 3D printed products (Cerdas et al., 2017). - Digital Twins in shared manufacturing (Wang et al., 2021; Glatt et al., 2021). - Self-organizing agents the idle or excess shared resources (Li & Jiang, 2021). - Cloud-based manufacturing services ecosystem (Zhang et al., 2019). - Blockchain to support trustable consumption reduction in collaborative and sustainable supply chains (Upadhyay et al., 2021). • ***Implementation of circular economy***: - Machine learning for circular manufacturing systems (Paraschos et al., 2022).

Similar to Tables 2 and 3 presents the elements of collaboration and added value regarding sustainability that are found in various works addressing the dimensions of “end-to-end engineering”, “smart products”, and the “creation of new business models”.

**Table 3:** **–** Examples of collaboration and sustainability aspects in the product/service dimensions

	Economic	Social	Environmental
**End-to-End Engineering**	• ***Reduce design cycles and costs***: - Using data collected from products and customers (Verhagen et al., 2015; Tao et al., 2019). • ***Add value to the product***: - Co-creation network (Yin et al., 2020).	• ***Co-creation & user innovation***: - Co-creation and user innovation methods (Rayna et al., 2015; Zheng et al., 2018; Yin et al., 2020). • ***Improve smart product design***: - Design / redesign smart product-service systems according to customer needs and wants (Zheng et al., 2018). - Ensuring product quality during production (Maleki et al., 2018).	• ***Design environmentally friendly solutions***: - Smart packaging design and life cycle assessment (Cabot et al., 2019). - Cloud-based ecosystem with different tools to design the product (including energy consumption reduction) (Zhang et al., 2019).
**Smart products / Digitalization**	• ***Reduce costs and increase efficiency***: - Extraction & analysis of products’ data along the supply chain to reduce costs (Zheng et al., 2018). - Creation of smart product symbiosis network (Yin et al., 2020). • ***Design better products***: - Framework to design/redesign better products (Zheng et al., 2018). - Collaborative networked Product Service System framework to increase value-added (Zhang et al., 2022).	• ***Increase smart product quality***: - Smart product-service quality (Maleki et al., 2018). • ***Co-creation & user innovation***: - Design framework / Service co-innovation in smart products (Zheng et al., 2018).	• ***Reduce environmental impact of product transportation***: - Continuous evaluation of the products’ conditions (Cabot et al., 2019). - Extraction & analysis of product data throughout the supply chain to reduce environmental impact (Zheng et al., 2018). - Design of Smart PSS in intelligent interoperable logistics (Pan et al., 2019). • ***Support circular economy***: - Smart products as enablers for circular economy (Alcayaga & Hansen, 2019). • ***Energy consumption and waste reduction***: - Data-driven framework for achieving sustainable smart product-service systems (Li et al., 2021). - Smart products characterized by service and sustainability concerns (Yin et al., 2020).
**New Business Models**	• ***Increase competitiveness***: - Sharing economy models (Li & Jiang, 2021; Wang et al., 2021)). • ***Reduce costs***: - Industrial symbiosis (Gao et al., 2020; Chen & Liu, 2021). • ***Increase resilience***: - Urban smart manufacturing and resilience (Sajadieh et al., 2022).	• ***Increase customer involvement***: - Stigmergic mass customization, co-creation, & co-design (Ogunsakin et al., 2021; Rayna et al., 2015; Turner et al., 2019). - Urban smart manufacturing and user involvement (Sajadieh et al., 2022). • ***Implement social manufacturing***: - Social manufacturing model (Jiang et al., 2016). - Hybrid value chains and social innovation (Budinich et al., 2007; Doherty & Kittipanya-Ngam, 2021). - Blockchain in social manufacturing (Leng et al., 2019).	• ***Reduce the environmental impact (energy consumption, waste reduction)***: - Circular economy-based business models (Rayna et al., 2015; Ansari et al., 2020). - Distributed SD printed manufacturing model and energy saving (Cerdas et al., 2017; Gupta et al., 2021). - Sharing economy to reduce waste and consumption (Li & Jiang, 2021; Wang et al., 2021). - Application of industrial symbiosis (Gao et al., 2020; Chen & Liu, 2021). - Global business sustainability beyond zero emissions (Svensson et al., 2016).

**Fig. 3:**
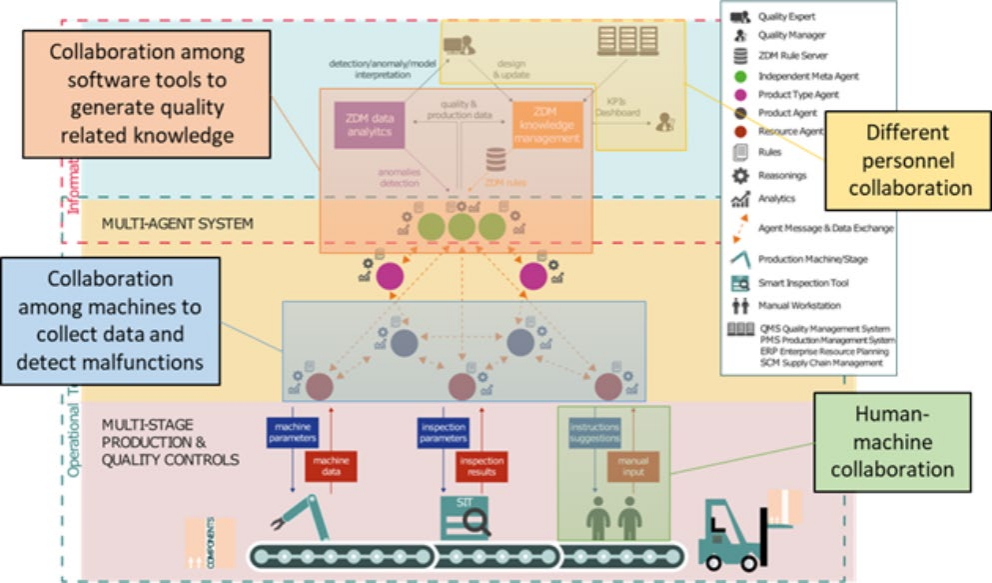
GO0DMAN High-Level Architecture and Collaborative Aspects. (adapted from (Angione et al., 2019))

**Fig. 4:**
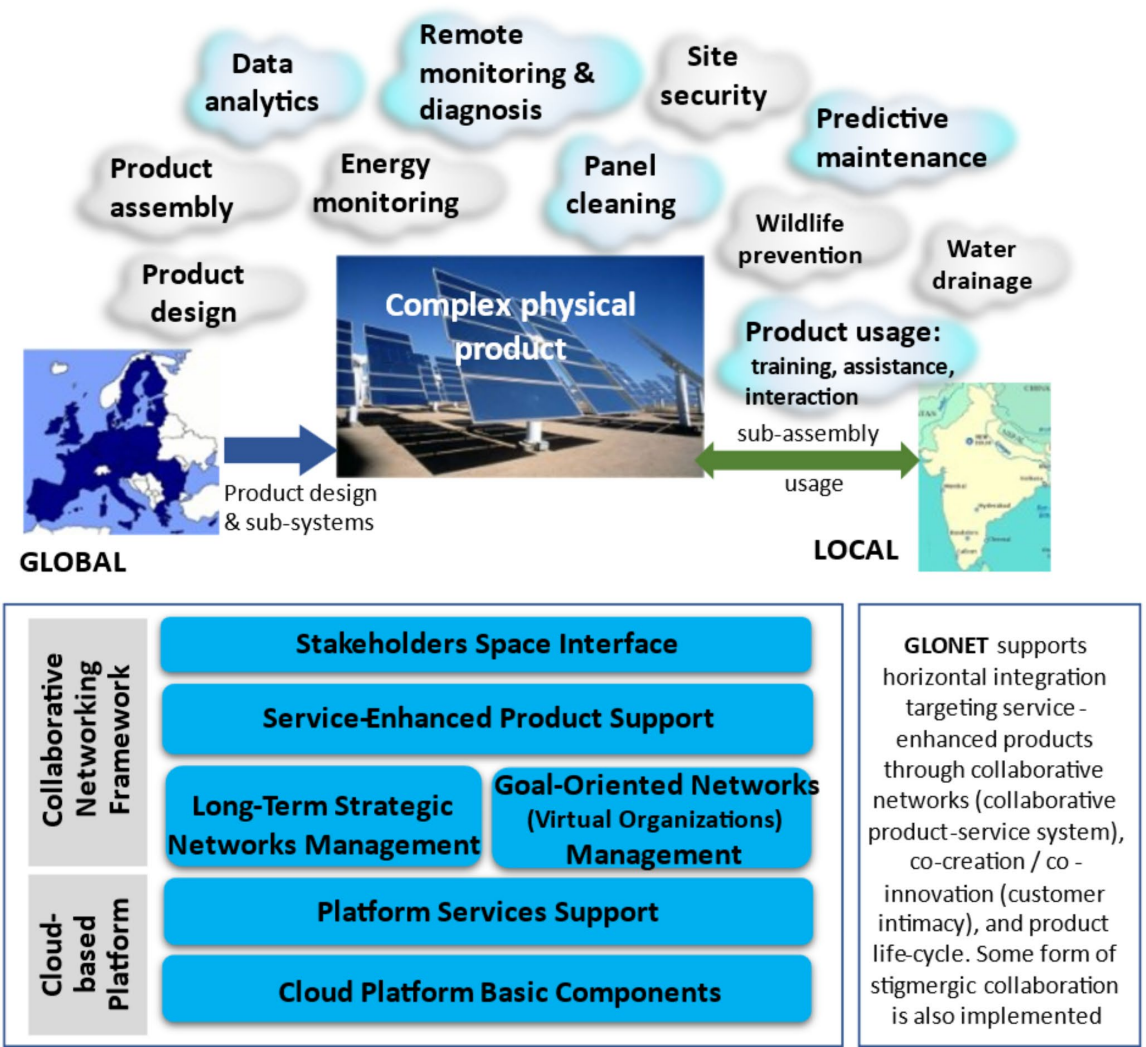
GLONET concept and High-Level Architecture

The results summarized in Tables 2 and 3 demonstrate that several efforts have already been made to develop more sustainable systems by combining Industry 4.0 practices and models and mechanisms from collaborative network. However, in most of the articles studied, the aspects of collaboration are usually not highlighted; nevertheless, the synergy between sustainable production and collaborative networks can be inferred. For example, two research projects in sustainable production in which our research center was involved are illustrated in Figs. 3 and 4, from which it is possible to observe aspects of collaboration at different levels of abstraction.

In the first example (Fig. 3), the GO0DMAN project had its main objective to use different tools, either hardware or software based, to create an environment capable of predicting quality problems related to parameterizations and deviations in the manufacturing process and the evolution of the product along the manufacturing line. Thus, several solutions have been developed that work together to deliver these forecasts. These tools focus on product inspection, process, and data extraction on the shop floor. In the cloud, we have software tools responsible for analyzing the collected data, creating forecast models, and interacting with the personnel responsible for process quality and maintenance. All these tools work in a collaborative ecosystem.

Regarding the second example, the GLONET project (Fig. 4) (Camarinha-Matos et al., 2017) is focused on supporting service-enhanced products in the areas of solar energy and smart buildings. The project illustrates a case of horizontal integration where a network of small and medium enterprises collaborate through a cloud-based platform to develop a product and its associated business services. The business services are collaboratively supported along the product life-cycle (which typically lasts 20–25 years).

Sustainability aspects in GLONET are reflected in the product itself (renewable energy support), the “*glocal* enterprise” concept, involving collaboration with local suppliers (close to the customer) and considering local specificities. Being solar plants complex and highly customized “products”, it is essential to involve the customer (and other local suppliers, the social pillar) in the process of creating new services and/or sub-systems, which corresponds to a of co-creation/co-innovation process that also requires the creation of temporary collaborative networks to develop these new systems/services.

From the cases studied, it can be seen that the facets of collaboration are already present in some cases of application of Industry 4.0 concepts to achieve sustainability. We can also notice that these facets appear more frequently at the level of the vertical and horizontal integration dimensions. Another interesting point is that emerging technologies, included in the “acceleration of the manufacturing” dimension, are particularly relevant for the efficient application of the collaboration aspects. However, it is important to note that the combination of manufacturing, sustainability, resilience, and collaborative networks is not always explicitly presented in the studied literature, but it is possible to infer its importance, as summarized in Tables 2 and 3. Regarding antifragility, although various examples in other domains can be found in the literature (Ramezani & Camarinha-Matos, 2020), it is still difficult to find practical cases in manufacturing.

## Measuring sustainability

Although the idea that sustainability is a major concern for modern manufacturing is gaining wide acceptance, its effectiveness needs to be measured, for which appropriate sustainability-related performance indicators must be established. As illustrated in Tables 4 and 5, some examples of efforts to measure sustainability in a context combining collaboration aspects and manufacturing and addressing economic, social and environmental concerns can already be found in the literature, although not yet fully developed.

The cases presented in Table 4, which address the manufacturing system perspective, mainly propose metrics and performance indicators to assess sustainability that are often borrowed from traditional manufacturing and supply chain performance indicators. Other works, rather than proposing specific indicators, offer some form of sustainability assessment framework. Regarding the collaboration perspective, a few initiatives can be found that highlight the importance of collaboration between various stakeholders to achieve better sustainability.

**Table 4:** – Examples of sustainability metrics/indicators in the manufacturing system dimensions

	Economic	Social	Environmental
**Vertical Integration**	• ***Economic sustainability metrics and indicators***: - Assessment at the production line level, although with few links to collaboration (Huang & Badurdeen, 2018). - Economic-related indicators and circularity index (Azevedo et al., 2010). • ***Economic sustainability assessment framework***: - Mapping the interconnections between technical and economic performance (Zhang et al., 2021b). - Evaluation of the quality of resilience in human-robot collaborative assembly (Lv et al., 2021).	• ***Social sustainability metrics and indicators***: - Assessment at the production line level, although with few links to collaboration (Huang & Badurdeen, 2018). - Economic-related indicators and circularity index (Azevedo et al., 2010). • ***Social sustainability assessment framework***: - Mapping the interconnections between technical and social performance (Zhang et al., 2021b). - Evaluation of the quality of resilience in human-robot collaborative assembly (Lv et al., 2021).	• ***Environmental sustainability metrics and indicators***: - Assessment at the production line level, although with few links to collaboration (Huang & Badurdeen, 2018). - Economic-related indicators and circularity index (Azevedo et al., 2010). • ***Environmental sustainability assessment framework***: - Mapping the interconnections between technical and environmental performance (Zhang et al., 2021b).
**Horizontal Integration**	• ***Economic sustainability metrics and indicators***: - Economic sustainability metrics for products and processes (Feng et al., 2010). - Economic indicators and sustainability index for supply chain (Salvado et al., 2015). - KPI dashboard for monitoring business performance in Virtual Enterprises (Hao et al., 2018). - Economic performance metrics in selecting sustainable suppliers (Sarkis & Dhavale, 2015). - Economic viability indicators in Industry 4.0 (for training) (Chaim et al., 2018). - Supply chain indicators to assess response to disruptions (Zidi et al., 2021). • ***Economic sustainability assessment framework***: - Methodology and metrics to evaluate sustainable manufacturing systems (Koren et al., 2018). - Multi-dimensional KPI space to control a supply chain’s trajectory according to risks and opportunities (Cerabona et al., 2020). - Method to study the sensitivity and fragility of a supply chain in face of risks or opportunities (Cerabona et al., 2021). - Sustainability and resilience criteria in supplier evaluation and selection (Zavala-Alcívar et al., 2020).	• ***Social sustainability metrics and indicators***: - Social sustainability metrics for products and processes (Feng et al., 2010). - Social performance metrics in selecting sustainable suppliers (Sarkis & Dhavale, 2015). - Social indicators and sustainability index for supply chain (Salvado et al., 2015). - Social sustainability indicators in Industry 4.0 (for training) (Chaim et al., 2018). - Supply chain indicators to assess response to disruptions (Zidi et al., 2021). • ***Social sustainability assessment framework***: - Methodology and social-oriented metrics to evaluate sustainable manufacturing systems (Koren et al., 2018). - Sustainability and resilience criteria in supplier evaluation and selection (Zavala-Alcívar et al., 2020). - “Organizational” and “sociocultural barriers” to sustainable manufacturing (Gupta et al., 2021a).	• ***Environmental sustainability metrics and indicators***: - Environmental sustainability metrics for products and processes (Feng et al., 2010). - Environmental performance metrics in selecting sustainable suppliers (Sarkis & Dhavale, 2015). - Environmental indicators and sustainability index for supply chain (Salvado et al., 2015). - KPI dashboard for monitoring environmental sustainability indicators in virtual enterprises (Hao et al., 2018). - Environmental sustainability indicators in Industry 4.0 (for training) (Chaim et al., 2018). - Performance indicators for industrial symbiosis network (Fraccascia et al., 2021). - Supply chain indicators to assess response to disruptions (Zidi et al., 2021). • ***Environmental sustainability assessment framework***: - Methodology and environment-oriented metrics to evaluate sustainable manufacturing systems (Koren et al., 2018; Glatt et al., 2021). - Assessment of the environmental performance of a supply chain based on balanced scorecard (Ferreira et al., 2016). - Sustainability and resilience criteria in supplier evaluation and selection (Zavala-Alcívar et al., 2020).
**Acceleration of Manufacturing**	• ***Economic sustainability metrics and indicators***: - Sustainability metrics regarding impact of Industry 4.0 technologies (Enyoghasi & Badurdeen, 2021). - Economic-oriented metrics for sustainable smart manufacturing (Abubakr et al., 2020). • ***Economic sustainability assessment framework***: - Sustainability in cybermanufacturing systems (Song & Moon, 2017). - Assess impact of Industry 4.0 technologies on sustainability (Beltrami et al., 2021; Kamble et al., 2020).	• ***Social sustainability metrics and indicators***: - Sustainability metrics regarding impact of Industry 4.0 technologies (Enyoghasi & Badurdeen, 2021). - Social-oriented metrics for sustainable smart manufacturing (Abubakr et al., 2020). • ***Social sustainability assessment framework***: - To assess ethical and sustainable performance in Industry 4.0 (Gupta et al., 2021a). - Sustainability in cybermanufacturing systems (Song & Moon, 2017). - Assess impact of Industry 4.0 technologies on sustainability (Beltrami et al., 2021; Kamble et al., 2020).	• ***Environmental sustainability metrics and indicators***: - Sustainability metrics regarding impact of Industry 4.0 technologies (Enyoghasi & Badurdeen, 2021). - Environment-oriented metrics for sustainable smart manufacturing (Abubakr et al., 2020). • ***Environmental sustainability assessment framework***: - To assess ethical and sustainable performance in Industry 4.0 (Gupta et al., 2021a). - Sustainability in cybermanufacturing systems (Song & Moon, 2017). - Assess impact of Industry 4.0 technologies on sustainability (Beltrami et al., 2021; Kamble et al., 2020).

Table 5 includes some examples of works contributing to sustainability metrics and indicators, and assessment frameworks focused on “end-to-end engineering”, “smart products, digitization”, and “new business models”. From this product/service perspective, the contributions identified are only preliminary approaches to measurement models, identification of benefits, and insights into their influence on sustainable performance. However, with respect to this perspective the number of developments is still very scarce.

**Table 5:** **–** Examples of sustainability metrics/indicators in the product/service dimensions

	Economic	Social	Environmental
**End-to-End ** **Engineering**	-	• ***Social sustainability metrics and indicators***: - Value co-creation metrics in service design (including some aspects of sustainability) (Botti et al., 2018).	
**Smart products ** **/ Digitalization**	• ***Economic sustainability metrics and indicators***: - Economic-oriented metrics to evaluate smart energy systems transition (Dincer & Acar, 2017).	• ***Social sustainability metrics and indicators***: - Social-oriented metrics to evaluate smart energy systems transition (Dincer & Acar, 2017).	• ***Environmental sustainability metrics and indicators***: - Environment-oriented metrics to evaluate smart energy systems transition (Dincer & Acar, 2017).
**New Business Models**	• ***Economic sustainability assessment framework***: - Impact of lean production and servitization on sustainable performance (Hao et al., 2021). - Performance measurement in servitization (Brax et al., 2021). - Quantitative analysis of the economic impact of Industry 4.0 enabled circular economy (Spaltini et al., 2021).	• ***Social sustainability assessment framework***: - Implications of servitization and digitalization in improvement of the organizational resilience and growth in healthcare manufacturing firms during the COVID-19 pandemic (Zhang & Qi, 2021).	• ***Environmental sustainability assessment framework***: - Impact of lean production and servitization on sustainable performance (Hao et al., 2021). - Quantitative analysis of the environmental impact of Industry 4.0 enabled circular economy (Spaltini et al., 2021). - A 3DR model (disassembly, deconstruction and resilience) to evaluate the level of the circularity of building and demolition industry (O’Grady et al., 2021).

It should be noted that this study includes only some illustrative examples and not an exhaustive portfolio of cases. Nevertheless, and despite the identified valuable attempts, it is apparent that there is still a lack of consolidated performance indicators to assess the benefits of collaboration in support of better manufacturing resilience and sustainability performance. For instance, the specific case of end-to-end engineering has received very little attention in this respect. For the other dimensions, it has only been possible to identify some assessment frameworks, without any proposal of concrete indicators.

## Research challenges

**Fig. 5:**
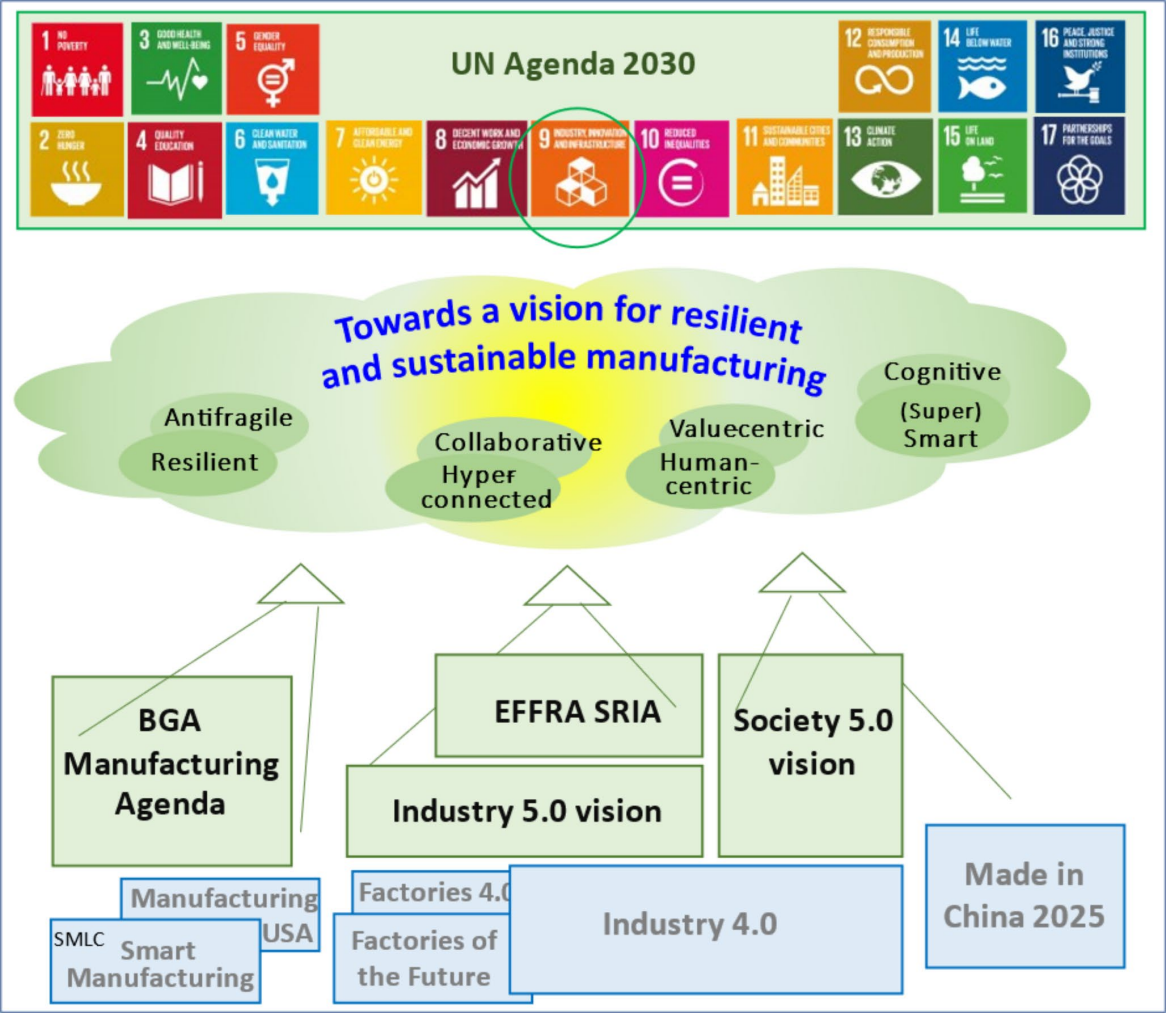
Some strategic research agendas contributing to resilient and sustainable manufacturing

The challenges of sustainable and resilient manufacturing are well reflected in various strategic research agendas promoted in different geographic regions. Influenced by the Strategic Development Goals of the UN 2030 Agenda (United Nations, 2015), most of these agendas are geopolitically motivated and, in some cases, constitute more of a policy guideline than a true research agenda, but are nevertheless representative of recent discussions and consolidation of ideas towards a vision for sustainable and resilient manufacturing systems (Fig. 5).

While Industry 4.0 is seen by many people as “technology-oriented”, more recently the term Industry 5.0 has started to be used, namely being pushed by the European Commission, as a complementary view that is more value-driven (Breque et al., 2021; i-Scoope, 2022; Xu et al., 2021). The vision is based on 3 pillars: an industry that is human-centric, sustainable, and resilient (i-Scoope, 2022). Aiming to leverage human creativity and agility in collaboration with smart machines and systems, Industry 5.0 shifts the focus, putting the human at the center (Maddikunta et al., 2022). Hence, smart / intelligent technologies should be designed to collaborate with the human, be resilient (e.g., business resilient, cyber resilient), and support sustainable practices. Distributed manufacturing, intelligent supply chains, and high levels of customization are also associated with the concept. In line with this vision, the EFFRA strategic research and innovation agenda (SRIA) “Made in Europe” (EFFRA, 2021) proposes a detailed plan around four main objectives: “Efficient, responsive and smart factories and supply chains”, “circular products & climate-neutral manufacturing”, “new integrated business, product-service and production” approaches, as well as “new use models”, and “human-centered and human-driven manufacturing innovation”. Although this agenda uses the terms “ecosystem” and “value network”, the aspects of collaborative networks are surprisingly not sufficiently highlighted, with the exception of the topic of “collaborative product-service engineering” and a few other references to “collaborative manufacturing”, “collaborative robotics”, “collaborative environments”, and “collaboration with AI”.

The manufacturing agenda elaborated by the BlueGreen Alliance (2020) is organized around five high-level pillars very focused on the transformation of the American industry, but where sustainability and clean economy concerns are clearly emphasized. However, it is more a kind of policy vision and not a true research agenda. As such, details about the technical approaches to be followed are lacking.

The Society 5.0 vision from Japan (H-UTokyo Lab, 2020; Broeckaert, 2022) shares several common goals with the vision for Industry 5.0, also emphasizing human-centric systems, smart systems, and balancing economic development with response to social issues, but going beyond manufacturing / industry and addressing society as a whole. In this plan one can find topics such as “human-centric approach to AI”, “advanced CPS and next generation ICT infrastructures”, “cybersecurity for all”, “decentralized and collaborative data platform”, and “promotion of the innovation and startup ecosystem”. In addition to the data platform, collaboration aspects can also be identified in manufacturing-related topics such as digital transformation and smart supply chains, smart manufacturing, and connected industries.

Another plan with geographical relevance is the Made in China 2025 agenda (Ling, 2018). This plan is, to some extent, inspired by the German Industry 4.0 initiative and aims to make China less dependent on advanced technology. While more contemporary to Industry 4.0 than to the current Industry 5.0 / Society 5.0 discussions, it also promotes digital manufacturing ecosystems, collaborative robotics, and includes some concerns for sustainable production and green manufacturing practices. The aspects of collaborative networks are not very explicitly emphasized in the agenda but looking at recent publications from the academic community in China, one can see a growing attention devoted to the topic.

As a result of the literature review and the findings of panels and focus groups discussions (e.g. from PRO-VE, DoCEIS, INDIN conferences in the last 3–4 years) summarized in previous sections (the baseline), and also taking into account the trends and limitations implicit in recent strategic research agendas and policy reports, it is possible to identify several topics that require further research to leverage synergies between collaborative networks and the vision of a resilient and sustainable manufacturing industry. Based on these gaps, a set of actions are proposed in complement to previous agendas / roadmaps, namely:

### Vertical integration

- At the shopfloor level, further rethink the organizational structure and design principles for CPS in terms of a collaborative ecosystem of smart entities, embedding sustainability metrics.
- In terms of supervision, move from a “control orientation” to a “collaborative orientation”, embedding the notions of sharing, coordination, negotiation and contracting between sub-systems, under a perspective of co-responsibility for sustainability.
- Further expand the notion of digital twin to more clearly embed the collaborative perspective and sustainability concerns.
- Extend the human-machine collaboration to a notion of a community of humans, smart machines, and intelligent systems (hybridization), guided by clear indicators of sustainability.

### Horizontal integration

- Extend existing reference models to address the inter-dependencies and interactions among multiple dynamic networks and develop corresponding governance models to better support the circular economy.
- Define clear metrics and indicators regarding co-responsibility in sustainability.
- Achieve a better understanding of issues in collaborative networks involving hybrid value systems, enhancing collaboration between manufacturing companies and the other societal actors.
- Achieve a better understanding of the principles of self-organization and co-evolution when constrained by performance indicators related to resilience and sustainability.
- Further explore the notions of resilience and antifragility in sustainable manufacturing under disruptive environments, in combination with the design of appropriate assessment indicators.

### Acceleration of manufacturing

- Explore the principles of collaborative ecosystems for better integration of new technology players with traditional manufacturing players.
- Find new ways to manage security and cyber-risks in hyper-connected environments, understand risk propagation, and develop counter-attack strategies, guided by sustainability principles.
- Further develop the smartness and sensing dimensions towards cognitive collaborative networks (distributed cognition and awareness) supporting sustainable manufacturing.
- Explore data-rich and big data environments and mechanisms to support new services and better decision making with respect to resilient and sustainable manufacturing. This should include special attention to fake data / data quality and how to deal with it collaboratively.

### End-to-end engineering

- Create a culture of collaboration through education and demonstration, enhancing multidisciplinary and interdisciplinary synergies.
- Further develop collaboration in open innovation and customer involvement (co-innovation and value co-creation) with new intellectual property models and sustainable customer intimacy / society-intimacy.
- Interlink the product life-cycle with collaborative models to better support the circular economy and industrial symbiosis.
- Explore AI and machine learning to better perceive societal trends.
- Develop appropriate sustainability metrics and indicators for this dimension.

### Smart product & digitalization

- Progress in designing smart products, integrating sustainability concerns.
- Improve distributed manufacturing models for smart products based on advanced and nature-inspired forms of collaboration such as stigmergy.
- Explore the potential of new exponential technologies to design smart products to better support traceability; explore distributed ledger technologies in support of sustainability co-responsibility.
- Support the addition of collaborative service models to products (service design and delivery).
- Explore product’s digital twins to assess value and impacts.
- Develop appropriate sustainability metrics and indicators for this dimension.

### New business models

- Further develop and apply collaboration principles in the circular economy and industry symbiosis.
- Evolve from enterprise-centric to collaborative business ecosystem-centric models.
- Further explore collaborative approaches in servitization and development of “glocal enterprises” for more sustainable systems.
- Further investigate the potential of networked additive manufacturing and micro-factories to support sustainability and “bring manufacturing back to cities”.
- Better understand the interactions between collaboration and competition in the context of sustainable business ecosystems.
- Identify and model emerging collaborative business models and assess their impact on sustainability through adequate metrics and indicators.

This list of topics is not intended to be a comprehensive research agenda, but rather to complement contemporary agendas and illustrate the wide range of research and development opportunities in manufacturing that are open when the ideas of collaborative networks and sustainability are combined.

## Conclusion

Sustainability is currently considered a major challenge for modern manufacturing systems, as reflected in recent strategic research agendas around the world. While the manufacturing sector has received renewed attention in recent years, as reflected in the proliferation of initiatives around Industry 4.0 and digital transformation, making such systems more sustainable remains a crucial challenge.

On the other hand, as manufacturing systems become increasingly intelligent, autonomous and interconnected, they reflect a kind of distributed intelligence/distributed cognitive systems. Thus, sustainability issues in this context benefit and need to be viewed from a distributed and collaborative perspective. Indeed, the effective achievement of sustainability requires the co-responsibility of multiple stakeholders. Moreover, we live in a time of frequent disruptive events that can drastically affect the operation of manufacturing systems. This raises the importance of considering resilience as a facet of sustainability. To this end, the synergies between collaborative networks and sustainable manufacturing need to be further explored.

In this study we have identified a number of proposed steps in this direction, both at the manufacturing system level and at the product/service/business model level. However, the collaboration aspects between all the entities present in these systems are not yet sufficiently considered and analyzed. Therefore, it is clear that despite several positive examples identified, there is a need to substantially pursue the exploration of synergies between the areas of manufacturing, sustainability and collaborative networks and to develop corresponding methodologies and evaluation indicators. To this end, a list of research topics is also suggested as an extension to the ongoing visions for Industry 5.0 and Society 5.0. The proposed list should not be seen as a complete research agenda in itself, but rather as a complement to contemporary agendas in manufacturing research. The central message of this proposal is that resilient and sustainable manufacturing can only be achieved through collaborative networks reflecting a notion of co-responsibility and engagement of all participating stakeholders for mutual benefit.
